# Case of Suppression of the Urine

**Published:** 1805-09-01

**Authors:** 


### To Dr. BRADLEY.

Sir,

FEW complaints are more painful in themselves, or more alarming in their consequences, than suppression of urine. The bladder will, in some instances, bear to be distended to a prodigious size with impunity, as we daily see in persons accustomed to swallow large quantities of liquor; while in others, if the effort to evacuate its contents be resisted but for a short time, the consequences are alarming. The greatest quantity of urine, which I recollect to have heard contained in the bladder, is mentioned in the 47th volume of the Journal de Medicine, where it is said, that sixteen pints of urine were drawn off by the catheter, in the space of two hours. The patient recovered, and in the course of two months the bladder had regained its proper tone. In some cases, when the distension has proceeded to a certain degree, Nature relieves herself for a time, though without removing the complaint, by exciting a flow of urine which deceives the bye-standers, and sometimes misleads the physician. This insidious case has been well described by Mr. Hey of Leeds, in his valuable Observations in Surgery; and the following instance, which occurred to me a short time ago, shews how cautious we ought to be in receiving the report of a nurse or a friend.

April 7, 1805, I was desired to visit the daughter of Philip Miller, living in this neighbourhood, and about ten years of age. I found she had, about a fortnight before, been affected with measles, from the effects of which she had not recovered. The cough continued severe after the eruption had disappeared; and in consequence of imprudent exposure to a cold wind, she was seized with symptoms of pneumonia. At the time of my visit, the cough was very troublesome, attended with much difficulty of breathing and acute pain in the chest. Her face was much flushed; her skin hot and dry. The pulse vibrated between 130 and 140 strokes in a minute, and was feeble; the tongue was brown and parched, with great thirst and restlessness. As she was costive, a few grains of calomel were ordered; a large blister was applied to the chest, and a mixture with spt. æth. nitros. was ordered occasionally, with an anodyne at night.

On

On the following day I found her in every respect much worse, though it seemed as if the blister had relieved the pain and extreme difficulty of breathing. She lay in a comatose state, and had much the appearance of a person in the last stage of typhus. Her pulse was above 140, and very feeble. The calomel had operated, but the stools and urine were passed involuntarily, and the latter in large quantity. As I considered this to be a hopeless case, and she lived at some distance, I did not see her for several days; but was informed by frequent messages, that no material alteration had occurred in the symptoms.

On the 17th, I was desired to visit her again; and to my astonishment, found her perfectly sensible, which had been the case since the preceding afternoon. In other respects, the symptoms were nearly as before, except that the cough and pneumonic symptoms had left her. She was still hot and restless; her pulse 160, and small; and she complained of very great thirst. Her thighs and labia pudendi were marked with black suffusions, and she observed that her belly felt hard though not painful. Upon examination I found a hard tumour extending from the pubis to above the umbilicus, and introducing the catheter I drew off three pints of dark coloured urine resembling coffee. I now found upon inquiry that she had made no water from five P. M. of Saturday, the day before my first visit, until four P. M. of Monday, some time before my second visit; but as she had experienced no uneasiness, and made no complaint, this circumstance was not noticed; from that time, however, the urine had continued to flow in such quantities that they could scarcely keep the bed dry. The child had experienced no pain from the distension, and felt no particular relief from the evacuation. Before my departure I explained the nature of the complaint, and desired that I might be informed if she continued unable to void her urine.

I heard nothing until the 19th, when I was told that she had made *plenty of water* since my visit, and had made at once about half a gill into the pot; but that she had been seized in the morning with a pain above the pubis, so acute as to cause her to scream aloud. I therefore desired that she might be put immediately into a warm bath, and if not relieved, that it might be repeated every three or four hours; ten drops of tinct. opii were ordered to be given, and a large blister to be applied to the hypogastrium. In the evening I found the pain considerably abated abated, but the bladder, notwithstanding the assertions of the parents, was distended nearly as much as before, and I drew off a full quart of dark coloured and extremely fœtid urine, containing a copious puriform sediment tinged with blood. Her pulse was still 160; she complained much of thirst, and had during the afternoon several fainting fits. The warm bath was ordered every night, and one of the following powders every three hours: ℞. Pulv. digital. gr. iij. pulv. uvæ ursi ʒj. ft. pulv. viij.

20th. In the evening I drew off with the catheter three large cups full and a half, amounting to about a pint, of dark coloured urine; the last half cup full appeared to be pure blood. In other respects, the symptoms were as before, except that she was perfectly free from pain. Her urine still flowed involuntarily.

21st. She passed a bad night, and experienced frequent attacks of severe pain above the pubis, with a painful desire to make water, which forced away several clots of blood, accompanied with gushes of bloody coloured urine. No tumour being perceptible in the hypogastric region, I did not introduce the catheter. Her pulse continued at 160; her features were much contracted, and the debility was so great as scarcely to bear removing into the warm bath, which was however ordered to be persisted in. The powders produced no nausea. For her thirst, which had somewhat abated, she took eagerly of milk, which had been her sole support during her illness.

22d. She appeared more composed this day, and had passed a better night; her pulse was about 140, and her skin felt cooler. The urine still continued to flow involuntarily, but about two ounces were passed at once of a pale red colour and offensive smell, but with somewhat less sediment.

24th. The urine has continued to flow involuntarily, but a few spoons full have been occasionally voided into the bed pan; it had no appearance of blood, and appeared nearly of the natural colour; the sediment also was much less. The warm bath was directed to be repeated every night, and the powders to be continued.

I did not see her until a week afterwards, when she had regained, in a considerable degree, the power of voiding her urine, which was perfectly natural in colour and had only a strong urinous smell. Her pulse had fallen to 100, and she appeared to have no other complaint than extreme debility and emaciation.

This

This child perfectly regained her health, though her recovery might, perhaps, rather be termed an escape. Nor do I feel myself perfectly free from censure, because I ought to have been more upon my guard; especially as I had long ago received a caution upon this subject from my late invaluable friend Dr. Clark,* of Newcastle, who some years ago read an account of two similar cases, accompanied with some valuable practical remarks, to the Medical Society in that town. These cases have unfortunately been lost or mislaid, but it is hoped they may yet be recovered, and probably appear, accompanied with some account of Dr. Clark, by a physician peculiarly fitted by former habits of intimacy to be his biographer.

I am, &c.

*July* 30, 1805. TYNICOLA.

---

* Dr. Clark was one of the best practical physicians in this kingdom, and was indebted solely to his own merit, which had overcome many obstacles, for the rank which he held in his profession. He possessed a profound knowledge of medicine, which his own extensive experience, and the study of the best antient and modern authors, aided by a retentive memory, had enabled him to acquire. He was accurate and diligent in his observation of diseases; cautious in forming his plan, but firm and decided in the execution. He was not much inclined to speculative notions, but omitted no means of storing his mind with practical facts; and when his professional engagements prevented him from indulging in a studious course of life, he never lost sight of the modern improvements in Medicine, though he was not easily induced to step aside from the path which experience had pointed out to him. Few physicians were more deservedly popular, or possessed in a greater degree the confidence of their patients; and it redounds still more to Dr. Clark's honour, that this popularity was acquired by no degrading arts; he despised the tricks of the Charlatan. For several years past I had repeated opportunities of meeting Dr. Clark in consultation, and of observing his practice; and it affords me a melancholy pleasure, in giving my mite of applause, to declare, that I always found him punctual in his appointments; anxious for the welfare of his patients; candid in giving his own opinion; and liberal in receiving that of others.

It was Dr. Clark's constant practice to take notes of every important case that occurred; and these, I have reason to believe, from a conversation in which he lamented his want of time to arrange them, he intended for the press. This valuable collection of facts will, I hope, in due time, be presented to the public, and though necessarily in an imperfect state, it will be received as a legacy worthy of the donor. To the above slight sketch I cannot refrain from adding the testimony of an excellent and amiable physician, who was almost daily in the habit of meeting Dr. Clark. "One object, (he observes) engaged his undivided attention through life, unceasing ardour in extending the narrow boundaries of the profession, by accuracy of observation and the vigorous application of remedies."

Copy

